# A frontal lobe mass in a 6‐year‐old girl

**DOI:** 10.1111/bpa.70132

**Published:** 2026-08-06

**Authors:** Francesca S. Vacca, Aaron Mochizuki, Smruti K. Patel, Blaise Jones, Somak Roy, Kirti Gupta

**Affiliations:** ^1^ Department of Pathology Cincinnati Children's Hospital Medical Center and University of Cincinnati Cincinnati Ohio USA; ^2^ Department of Oncology Cincinnati Children's Hospital Medical Center and University of Cincinnati Cincinnati Ohio USA; ^3^ Division of Pediatric Neurosurgery Cincinnati Children's Hospital Medical Center and University of Cincinnati College of Medicine Cincinnati Ohio USA; ^4^ Department of Radiology and Medical Imaging Cincinnati Children's Hospital Medical Center and University of Cincinnati Cincinnati Ohio USA

**Keywords:** ALK‐positive histiocytosis, Dural mass

1

BOX 1 **Virtual glass slide box**

Access at https://isn‐slidearchive.org/?col=ISN&fol=Archive&file=D‐4168532_HE_wsi.svs.

## 2 CASE HISTORY AND IMAGING

**FIGURE 1:**
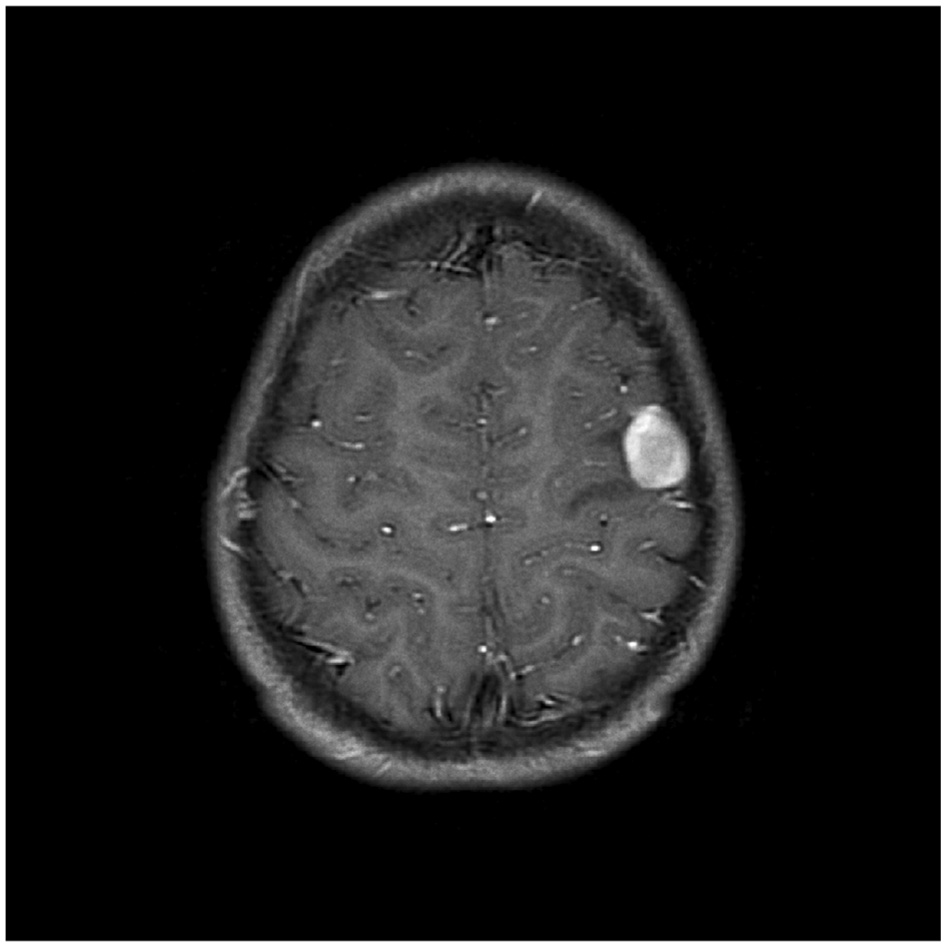
Axial T1‐weighted magnetic resonance (MR) image with fat‐saturation and intravenous gadolinium shows an avidly enhancing ovoid tumor in the periphery of the left frontal lobe with adjacent vasogenic edema.

A 6‐year‐old girl presented with 3 weeks of right‐hand weakness, shaking, and a single generalized seizure. Electroencephalogram showed epileptiform discharges in the left central region. Magnetic resonance imaging (MRI) revealed an enhancing mass, 1.7 × 1.3 cm, in the periphery of the left frontal lobe with moderate vasogenic edema in the adjacent white matter (Figure 1). The mass showed homogeneous and diffuse enhancement with peripherally pronounced diffusion restriction. Additional MRI findings included a Chiari I malformation, spinal syrinx extending from C2 to C7, Klippel‐Feil fusion anomaly of C6–C7, and a venous vascular anomaly of the left frontoparietal junction. She underwent a left‐sided stereotactic frontal craniotomy for resection of the lesion. Upon dural opening, it was noted that the lesion did not have any dural attachment and found to be cortically based. Using microsurgical techniques, the lesion was carefully separated from the underlying brain and gross total tumor resection was achieved. The patient recovered without complication.

## 3 HISTOLOGY

**FIGURE 2:**
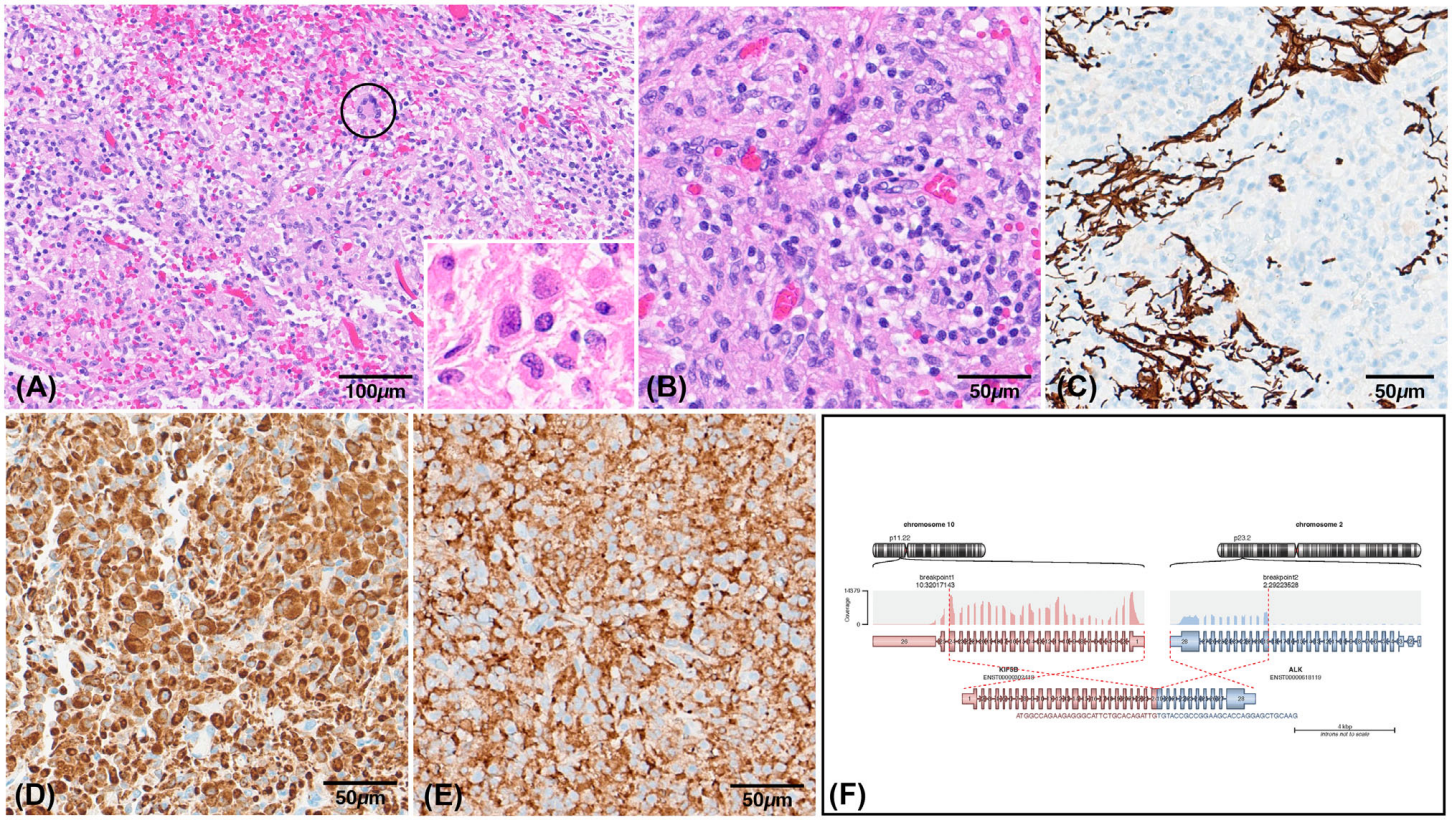
(A) Low magnification, sheets of oval, epithelioid, plump fusiform cells and a rare Touton giant cell (circle); inset, nuclear convolutions and grooves. (B) High magnification, tumor cells with vacuolated cytoplasm admixed with few mature lymphocytes. (C) Tumor cells are negative for GFAP (C) and show strong immunoreactivity for C163 (D) and ALK (E). (F) Genomic representation of *KIF5B::ALK* fusion with breakpoints involving exon 24 of *KIF5B* and exon 19 of *ALK*.

Sections demonstrated an infiltrative lesion composed of large, ovoid, epithelioid cells, and plump, fusiform cells arranged in sheets, within a pale, fibrillary background (Box 1). The lesion infiltrated into the adjacent brain parenchyma, with prominent perivascular aggregates of plump cells. The cells exhibited mild pleomorphism with irregular, convoluted, or grooved nuclei and indistinct nucleoli. Scattered cells with vacuolated cytoplasm were present, along with rare Touton giant cells and binucleate forms (Figure 2A,B). The lesion was supported by a network of delicate capillaries. No mitotic figures, significantly atypical nuclei, or areas of necrosis were identified. Focal infiltrates of mature lymphocytes were also identified. The adjacent cortex showed a mixture of tumor cells and scattered cortical neurons in a gliotic background. Reticulin stain highlighted prominent fibers around the vessels accentuating the perivascular localization of lesional cells. The lesional cells were strongly and diffusely positive for CD68 and CD163 (Figure 2D), while negative for GFAP (Figure 2C), NeuN, Neurofilament, Olig‐2, Synaptophysin, EMA, BCOR, CD1a, BRAF (VE1), and CD99. INI‐1 was retained. A subset of tumor cells were positive for ALK (cytoplasmic and Golgi‐dot pattern, Figure 2E), cyclin‐D1, Factor XIIIa, and p‐ERK. The Ki‐67 index was low, 2–3%, with strongest staining near lymphocytic infiltrates. NeuN highlighted scattered, mature, cortical neurons in the adjacent brain parenchyma. Neurofilament revealed marked axonal filaments within the lesional cells with diffuse positivity in the adjacent parenchyma, supporting an infiltrative pattern.

Hybrid‐capture based targeted next generation sequencing analysis identified a *KIF5B::ALK* fusion in this tumor with breakpoints involving exon 24 of *KIF5B* and exon 19 of *ALK*. RNAseq data demonstrated disproportionately higher expression of the tyrosine kinase domain in contrast to the extracellular domain of the *ALK* transcript consistent with oncogenic *ALK* activation (Figure 2F).

## 4 FINAL DIAGNOSIS

ALK‐positive histiocytosis.

## 5 DISCUSSION

ALK‐positive histiocytosis is a rare neoplasm that classically presents in very young children with multisystem involvement [1, 2]. It is also described as localized disease and in patients of all ages [1, 3]. The most common sites of involvement include the central nervous system (CNS), lungs, bone, liver, spleen, bone marrow, and skin [2, 3]. CNS involvement occurs in approximately half of reported cases, and typically involves intra‐dural, extra‐axial regions including the cavernous sinus, leptomeninges, and cranial nerves [2, 3]. The clinical presentation is diverse, and dependent upon tumor location. The classic presentation is an infant with abdominal distension, anemia, thrombocytopenia, and additional signs of localized disease such as seizure, focal neurologic deficit, or skin lesions [1, 2, 3].

Despite the variable clinical presentations, the histologic features remain relatively consistent: sheets of epithelioid histiocytes with irregular nuclear contours, grooved nuclei, fine chromatin, small nucleoli, occasional multinucleation, and abundant, pale, eosinophilic, and sometimes foamy cytoplasm [1, 2, 3]. Touton‐type giant cells are also often present [1, 2]. The tumor cells are positive for CD68, CD163, and show variable S100 expression, while negative for BRAF V600E, CD1a, and Langerin [2]. Immunohistochemical staining for ALK‐1 is variable with a cytoplasmic, Golgi dot‐like or rarely membranous patterns, but practically never nuclear, as shown by molecularly defined cases [2]. Immunostain can be focal and weak [2].

Molecular evidence of ALK‐fusion is diagnostic. Multiple discrete ALK‐fusions have been described, however *KIF5B::ALK* is the most common fusion, present in 27 of 39 cases in the largest published series [2]. *KIF5B::ALK* is rarely documented independently of ALK‐positive histiocytosis [3]. The *ALK* gene encodes a receptor tyrosine kinase, while *KIF5B* encodes a widely expressed microtubular transport protein [3]. Fusion involves the kinase domain of *ALK* and the kinesin dimerization region of *KIF5B*, leading to a chimeric protein that most likely functions as a constitutively active kinase [3]. This theory is supported by good patient responses to ALK‐inhibitor treatment [2, 3].

This case is a classic example of ALK‐positive histiocytosis, an uncommon entity that should be considered in pediatric cases presenting with dural‐based or extra‐axial masses or concomitant systemic disease. Accurate diagnosis is essential to ensure the timely initiation of targeted therapy.

## AUTHOR CONTRIBUTIONS

*Conceptualization*: KG and SR. *Data analysis and interpretation*: FV, AM, SKP, BJ, SR, and KG. *Drafting*: FV and KG. *Critical review/editing*: KG and SR. *Supervision*: KG. All authors have approved the final manuscript.

## CONFLICT OF INTEREST STATEMENT

The authors declare no conflicts of interest.

## ETHICS STATEMENT

This work is waived from IRB approval because of the status as a case report.

## PATIENT CONSENT STATEMENT

Consent was obtained from the patient's legal guardian.

## Data Availability

The data supporting the findings of this case are available on reasonable request from the corresponding author.
